# Hypertensive disorders of pregnancy and perinatal outcomes: two prospective cohort studies of nulliparous women in India and Tanzania

**DOI:** 10.1136/bmjgh-2024-016339

**Published:** 2025-07-10

**Authors:** Andrea B Pembe, Pratibha Dwarkanath, Amani Kikula, John Michael Raj, Nandita Perumal, Heavenlight A Paulo, Rajalakshmi M, Christopher P Duggan, Honorati M Masanja, Nandini Chopra, Mary M Sando, Tinku Thomas, Cara A Yelverton, Alfa Muhihi, Anura V Kurpad, Wafaie W Fawzi, Blair J Wylie, Christopher R Sudfeld

**Affiliations:** 1 Muhimbili University of Health and Allied Sciences, Dar es Salaam, Dar es Salaam, Tanzania, United Republic of; 2 St John’s Research Institute, Bangalore, Karnataka, India; 3 Institute of Tropical Medicine, Antwerp, Belgium; 4 University of Antwerp, Antwerp, Belgium; 5 St John’s Medical College, Bangalore, Karnataka, India; 6 Harvard T H Chan School of Public Health, Boston, Massachusetts, USA; 7 University of South Carolina, Columbia, South Carolina, USA; 8 Boston Children’s Hospital, Boston, Massachusetts, USA; 9 Harvard Medical School, Boston, Massachusetts, USA; 10 Ifakara Health Institute, Dar es Salaam, Tanzania, United Republic of; 11 Africa Academy for Public Health, Dar es Salaam, Tanzania, United Republic of; 12 Beth Israel Deaconess Medical Center, Boston, Massachusetts, USA

**Keywords:** Obstetrics, Hypertension, Child health, Cohort study

## Abstract

**Introduction:**

Hypertensive disorders of pregnancy (HDP) have been linked with increased risk for maternal and offspring complications in high-income settings. However, in resource-limited settings, studies with robust measurement of HDP, including severity and timing, and perinatal outcomes are limited.

**Methods:**

We analysed data from two prospective cohorts of nulliparous women in India (n=10 570 pregnancies) and Tanzania (n=10 299 pregnancies) who were enrolled in calcium supplementation trials and had blood pressure and proteinuria assessments throughout pregnancy and at the time of labour and delivery. Generalised estimating equations were used to assess the relationship between HDP severity categories (gestational hypertension, preeclampsia without severe features and preeclampsia with severe features) and timing of HDP onset (early-onset <34 weeks vs late-onset ≥34 weeks gestation) with adverse perinatal outcomes.

**Results:**

The cumulative incidence of HDP was 3.7% and 4.5% in the India and Tanzania cohorts, respectively. All HDP severity categories were associated with a significantly higher risk for perinatal death in both cohorts (p values<0.05). Pregnancies complicated by pre-eclampsia with severe features had the largest magnitude of increased risk for perinatal death as compared with pregnancies without an HDP (India RR 8.60; 95% CI 5.90 to 12.53; Tanzania RR 4.05; 95% CI 2.91 to 5.66). In both cohorts, pre-eclampsia with and without severe features, but not gestational hypertension, was associated with increased risks for preterm birth and low birth weight. Pregnancies with early-onset HDP had a high absolute risk of perinatal death (India 25.6% and Tanzania 36.9%), and the risk was markedly increased as compared with late-onset HDP (India RR 4.79; 95% CI 2.68 to 8.54; Tanzania RR 5.79; 95% CI 3.56 to 9.41).

**Conclusion:**

HDP were differentially associated with risks for perinatal outcomes by severity and timing of onset. Interventions that prevent, reduce the severity or delay the onset of HDP may improve perinatal outcomes in resource-limited settings.

**Trial registration number:**

ClinicalTrials.gov number, NCT03350516

**WHAT IS ALREADY KNOWN ON THIS TOPIC**

- Hypertensive disorders of pregnancy (HDP), which affect 5%–10% of pregnancies, remain a leading cause of maternal death globally.
- HDP may also affect the risk for a range of adverse offspring outcomes, including perinatal death, low birth weight, preterm birth and small-for-gestational age births, but data are very limited in the context of LMICs where most adverse perinatal outcomes occur globally.

**WHAT THIS STUDY ADDS**

- We conducted two prospective cohort studies of nulliparous pregnancies in India and Tanzania to examine the association of severity and timing of HDP with perinatal outcomes. In both cohorts, gestational hypertension, pre-eclampsia without severe features and pre-eclampsia with severe features were each associated with an increased risk of perinatal death as compared with pregnancies without an HDP. Pre-eclampsia with severe features had the largest magnitude of risk for perinatal death.
- Pre-eclampsia with and without severe features was both independently associated with increased risk for preterm birth and low birth weight in both cohorts. Both were also associated with a greater risk for SGA births in the Tanzania cohort.
- Early-onset HDP were associated with a strong magnitude of an increased risk for perinatal death, stillbirth, preterm birth and lower birthweights as compared with late-onset HDP in both cohorts.

**HOW THIS STUDY MIGHT AFFECT RESEARCH, PRACTICE OR POLICY**

- In the context of resource-limited settings, HDP are associated with an increased risk of adverse perinatal outcomes, with early-onset and more severe forms of HDP carrying a strikingly high absolute risk of perinatal death.
- Implementing effective interventions that prevent, reduce the severity or delay the onset of HDP may improve perinatal outcomes in LMICs.

## Introduction

 Hypertensive disorders of pregnancy (HDP), which affect 5%–10% of pregnancies, include chronic hypertension, gestational hypertension, pre-eclampsia/eclampsia and pre-eclampsia superimposed on chronic hypertension.^1^,^3^ The severity of HDP has been shown to differentially affect the risk of maternal complications, including a higher risk of longer-term maternal cardiovascular events.^4^,^6^ HDP may also differentially affect the risk for a range of adverse offspring outcomes, including perinatal death, low birth weight (LBW), preterm birth and small-for-gestational age (SGA) births.^7 8^ However, few prospective cohort studies with robust measurement of blood pressure and proteinuria in pregnancy and around the time of delivery have examined the relationship of HDP with birth outcomes in the context of low- and middle-income countries (LMICs), where the majority of adverse perinatal outcomes occur globally.^9 10^

A recent systematic review and meta-analysis of pregnancy cohorts that examined the relationship between HDP and adverse perinatal outcomes identified a large number of studies from high-income settings, but only five pregnancy cohorts were identified from South Asia and Sub-Saharan Africa.^7^ In addition, few studies have compared perinatal outcomes for early-onset (<34 weeks gestation) to late-onset HDP (<34 weeks gestation) with no data, to the best of our knowledge, in LMICs. Infants born preterm have a higher absolute risk of mortality in LMICs as compared with high-income country settings, and therefore, pregnancies with early-onset HDP may be associated with a greater magnitude of mortality risk in LMICs.^11^ In addition, the cohort studies conducted in South Asia and Sub-Saharan Africa primarily analysed administrative datasets or clinical records which may lead to significant misclassification of HDP.^12 13^ Prospective cohort studies with robust assessments of HDP throughout pregnancy are therefore needed to assess and quantify the association of HDP severity and timing of onset with perinatal outcomes in Sub-Saharan Africa and South Asia.

We conducted two prospective cohort studies of pregnancies in India and Tanzania to assess the relationship of HDP severity and timing of onset with perinatal outcomes. We hypothesised that more severe forms of HDP would be strongly associated with an increased risk of adverse perinatal outcomes and that early-onset HDP would also confer a greater risk as compared with late-onset HDP.

## Methods

We conducted two prospective cohort studies of pregnant women and their offspring in India and Tanzania who were enrolled in harmonised non-inferiority trials of low dose as compared with standard high-dose calcium supplementation in pregnancy.^14 15^ The trial in India was conducted in Bangalore from November 2018 to December 2022, while the trial in Tanzania was conducted in Dar es Salaam from March 2019 to December 2022. Both trials had the same inclusion criteria and enrolled nulliparous pregnant women<20 weeks gestation who intended to stay in the study area until 6 weeks postpartum and gave written informed consent. Exclusion criteria for the trials were (i) a history, signs or symptoms of nephrolithiasis; (ii) a prior diagnosis of a parathyroid disorder or a thyroidectomy; or (iii) a disease that required digoxin, phenytoin, or tetracycline therapy. For this analysis, we also excluded participants with chronic hypertension, defined as the use of antihypertensive medications or a measurement of hypertension before 20 weeks gestation, given the very low prevalence (less than 1% in both trials). We therefore did not have statistical power to assess the relationship of chronic hypertension or pre-eclampsia superimposed on chronic hypertension with perinatal outcomes.

The data collection and follow-up procedures of the trials are fully described elsewhere.^14 15^ Briefly, participants had follow-up clinic visits each month during pregnancy, at delivery and at 6 weeks postpartum. At the baseline visit, pregnant women were administered a questionnaire to assess that sociodemographic characteristics, height and weight were measured and haemoglobin concentrations were assessed (India, HemoCue Hb 301; Tanzania, HemoCue Hb 201 or HemoCue AB, Ängelholm, Sweden). Pregnant women also underwent obstetric ultrasound as part of the research protocol with measurements of foetal biometry. The best obstetric estimate approach was used to estimate gestational age (methods detailed in the trial protocol).^14^

Standardised and harmonised blood pressure and urine protein assessment procedures were implemented in both trials, and data were collected at baseline, monthly pregnancy follow-up and delivery visits. Blood pressure was obtained twice in the seated position at 30 s intervals. Participants with an average systolic blood pressure of 140–159 millimetres of mercury (mm Hg) systolic or diastolic blood pressure of 90–109 mm Hg were scheduled for a follow-up blood pressure assessment 1 hour to 1 week later. Participants with an average blood pressure in the severe range (systolic blood pressure ≥160 mm Hg or a diastolic blood pressure ≥110 mm Hg) had blood pressure reassessed at 1 min. Blood pressures at the time of delivery were assessed by study team members or abstracted from medical records. In addition, at all study visits from pregnancy to delivery, urine was collected and assessed for the presence of protein by dipstick. At any time during the trials, all participants identified as having elevated blood pressure or proteinuria were referred for medical care.

Hypertension was defined as a systolic blood pressure ≥140 mm Hg or a diastolic blood pressure ≥90 mm Hg on two occasions at least 1 hour apart. Severe hypertension was defined by a systolic blood pressure ≥160 mm Hg or a diastolic blood pressure ≥110 mm Hg on two occasions at least 1 min apart in pregnancy or on one occasion at labour or delivery.^14 15^ Proteinuria was a dipstick reading of greater or equal to 1+. Chronic hypertension was defined by the use of anti-hypertensive medications before 20 weeks gestation or hypertension detected before 20 weeks gestation; these participants were excluded from this analysis. Gestational hypertension was defined as new-onset hypertension after 20 weeks gestation without proteinuria. Pre-eclampsia without severe features was defined by new-onset hypertension (non-severe range) with proteinuria after 20 weeks gestation. Pre-eclampsia with severe features was defined by severe gestational hypertension with or without proteinuria; eclampsia; end-organ dysfunction; HELLP syndrome (haemolysis, elevated liver enzymes and low platelet count); pulmonary oedema; or new-onset central nervous system or visual symptoms.^14 15^ Participants were categorised by the most severe form of HDP documented. Treatment for HDP was provided by non-study clinic staff per the standard of care in each setting.

The vital status of births was ascertained from direct observation, medical records and/or verbal autopsy. Infant birth weight was measured using standardised and calibrated digital scales or abstracted from medical records. Infant vital status was assessed at a study visit at 6 weeks of age. Stillbirth was defined as births after 28 weeks gestation without evidence of breathing or any signs of life. Perinatal deaths included stillbirths and early neonatal deaths (≤ 7 days after birth). Preterm birth was defined as a live birth <37 weeks gestation, LBW as a live birth with a birthweight <2500 grams and SGA as a live birth <10^th^ percentile for gestational age and sex using the INTERGROWTH-21^st^ standards.^16^

### Statistical analysis

Statistical analyses were conducted separately for the India and Tanzania cohorts due to potential differences in the relationship of HDP with adverse perinatal outcomes. The analytic study populations were aligned for both cohorts and excluded pregnant participants with chronic hypertension, women who were determined to not be pregnant on ultrasound, women with a miscarriage or medically induced abortion before 28 weeks gestation and pregnant women with an unknown birth outcome (withdrawal of consent, death, or loss to follow-up).

Pregnant women were classified into four categories: no HDP (reference group), gestational hypertension, pre-eclampsia without severe features and pre-eclampsia with severe features. Pregnancies were classified by the most severe category of HDP reached during pregnancy, and therefore each pregnant woman only contributed to one of the HDP categories. For HDP categories, we described the gestational age at the first diagnosis of hypertension, at the time of the most severe HDP diagnosis and at the time of delivery. We then evaluated the association of HDP categories with the outcomes of perinatal death, stillbirth, gestational age at delivery (continuous outcome), preterm birth, birth weight (continuous outcome), LBW and SGA. Generalised estimating equations (GEE) with log links were used to assess relative risks for binomial outcomes, while the identity link was used to estimate mean differences for continuous outcomes. Compound symmetric correlation matrices were used to account for multiple gestations. Multivariable models were similarly constructed for both cohorts and included adjustment for baseline maternal age (18–24, 24–29, ≥30 years), education (India (no formal education, primary/middle school, high school, pre-university college/diploma or advanced); Tanzania (no formal education, primary, secondary)), wealth quintile (based on a principle component analysis of household assets), height (<150, 150.0 to 154.9, ≥155 cm), body mass index (BMI) (<18.5, 18.5–24.9, 25.0–29.9, ≥30 kg/m^2^), haemoglobin concentration (non-anaemic ≥11.0 g/dL, mild anaemia 10.0–10.9 g/dL, moderate or severe anaemia <10.0 g/dL), HIV status (Tanzania only: positive or negative) and randomised regimen (500 or 1500 mg calcium). The missing indicator method was used to retain data when covariate missingness was >1%. Statistical analyses were performed with the use of SAS software, V.9.4 (SAS Institute). An author reflexivity statement was completed (online supplemental file 1).

### Patient and public involvement

Patients and/or the public were not involved in the design, conduct, reporting, or dissemination plans of this research.

## Results

The flow diagrams for the analytic study populations for the India and Tanzania cohorts are presented in online supplemental figures 1 and 2, respectively. For the India cohort, 10 506 pregnancies with 10 570 foetuses were included, while the Tanzania cohort included 10 299 pregnancies with 10 467 foetuses. Baseline characteristics of pregnant participants in the India and Tanzania cohorts are presented in table 1. In both cohorts, most participants were between 18 and 24 years of age, and ~30% had a baseline BMI >25 kg/m^2^ and nearly half of the women had anaemia.

**Table 1:** Baseline characteristics of nulliparous pregnant women without chronic hypertension included in the India and Tanzania cohorts*

	India(N=10 506)n (%)	Tanzania(N=10 299)n (%)
Maternal age		
18–24 years	6533 (62.2%)	7324 (71.1%)
25–29 years	3010 (28.7%)	2364 (23.0%)
30+ years	963 (9.2%)	611 (5.9%)
Maternal education		
No formal education	315 (3.0%)	165 (1.6%)
Primary	388 (3.7%)	3352 (32.5%)
Secondary or higher	9803 (93.3%)	6782 (65.9%)
Maternal height <150.0 cm	2315 (22.0%)	3441 (33.4%)
Body mass index (BMI)		
<18.5 kg/m^2^	1735 (16.5%)	842 (8.2%)
18.5–24.9 kg/m^2^	5490 (52.3%)	5998 (58.2%)
25.0–29.9 kg/m^2^	2328 (22.2%)	2390 (23.2%)
≥30.0 kg/m^2^	953 (9.1%)	1069 (10.4%)
Haemoglobin concentration		
No anaemia ≥11.0 g/dL	5784 (55.2%)	6033 (58.6%)
Mild anaemia 10.0–10.9 g/dL	3285 (31.3%)	2892 (28.1%)
Moderate or severe anaemia <10.0 g/dL	1415 (13.5%)	1374 (13.3%)
Living with HIV	–	178 (1.7%)
Randomised calcium regimen		
500 mg calcium	5248 (50.0%)	5179 (50.3%)
1500 mg calcium	5258 (50.0%)	5120 (49.7%)

* Analytic study population excludes pregnant women who had chronic hypertension, were determined not pregnant by ultrasound, withdrew consent prior to delivery, died in pregnancy, were lost to follow-up prior to delivery or had a pregnancy loss before 28 weeks gestation.

Table 2 describes the timing of onset and delivery outcomes for HDP cases stratified by site. In the India cohort, the cumulative incidence of any HDP, excluding chronic hypertension, was 3.7% (386 women with HDP among 10 506 pregnancies, 95% CI 3.3% to 4.1%), and among pregnant women with an HDP, the percentages with gestational hypertension, pre-eclampsia without severe features and pre-eclampsia with severe features were 17.1% (n=66 among 386 pregnancies with an HDP), 46.6% (n=180 among 386 pregnancies with an HDP) and 36.3% (n=140 among 386 pregnancies with an HDP), respectively. In the Tanzania cohort, the cumulative incidence of any HDP was 4.5% (464 women with HDP among 10 299 pregnancies, 95% CI 4.1% to 4.9%), and among pregnancies with an HDP, 34.7% had gestational hypertension (n=161 among 464 pregnancies with an HDP), 25.4% pre-eclampsia without severe features (n=118 among 464 pregnancies with an HDP) and 39.9% had pre-eclampsia with severe features (n=185 among 464 pregnancies with an HDP). In terms of timing of onset of the first hypertensive diagnosis, in the India cohort, 31.1% (120 of 386 HDP cases) of HDP cases were early-onset and diagnosed prior to 34 weeks, while only 16.8% (78 of 464 HDP cases) of HDP cases were early-onset in Tanzania. The proportion of early-onset cases was similar among gestational hypertension and preeclampsia without severe features cases between the cohorts; however, early-onset was more common among preeclampsia with severe features cases in India (63 early-onset among 140 pre-eclampsia with severe feature cases, 45.0%) compared with Tanzania (33 early-onset among 185 pre-eclampsia with severe feature cases, 17.8%). In both countries, ~90% of pregnancies were delivered within 48 hours of the most severe HDP category diagnosis. Labour induction was more common in India compared with Tanzania across HDP categories.

**Table 2:** Descriptive characteristics of the timing of diagnosis and delivery by hypertensive disorder of pregnancy category in the India and Tanzania cohorts

	Gestational hypertension*	Pre-eclampsia without severe features*	Pre-eclampsia with severe features*
*India*	(*n=66 pregnancies*)	(*n=180 pregnancies*)	(*n=140 pregnancies*)
Median (Q1, Q3) gestational age at diagnosis of first HDP (including less severe categories)	38.3 weeks(35.7, 39.4)	36.8 weeks(33.8, 38.7)	35.8 weeks(32.7, 38.5)
% Early onset at first HDP diagnosis (<34 weeks)	10/66 (15.2%)	47/180 (26.1%)	63/140 (45.0%)
Median (Q1, Q3) gestational age at diagnosis of most severe HDP category	†	37.8 weeks (35.5, 39.6)	36.6 weeks (34.4, 38.9)
% Concurrent diagnosis of the first and most severe HDP category	†	141/180 (78.3%)	106/140 (75.7%)
Median (Q1, Q3) gestational age at delivery	39.0 weeks (37.1, 39.7)	38.3 weeks (36.9, 39.7)	36.8 weeks (35.0, 39.0)
% Delivery within 48 hours of the most severe HDP category diagnosis	47/66 (71.2%)	134/180 (74.4%)	125/140 (89.3%)
% Induced labour‡	25/63 (39.7%)	58/158 (36.7%)	54/123 (43.9%)
*Tanzania*	(*n=161 pregnancies*)	(*n=118 pregnancies*)	(*n=185 pregnancies*)
Median (Q1, Q3) gestational age at diagnosis of first HDP (including less severe categories)	38.7 weeks (36.7, 40.3)	37.5 weeks (34.9, 39.1)	38.6 weeks (35.7, 39.9)
% Early onset of first HDP (<34 weeks)	24/161 (14.9%)	21/118 (17.8%)	33/185 (17.8%)
Median (Q1, Q3) Gestational age at diagnosis of most severe HDP category	†	37.7 weeks (35.6, 39.1)	38.6 weeks(36.0, 39.9)
% Concurrent diagnosis of the first and most severe HDP category	†	114/118 (96.6%)	177/185 (95.7%)
Median (Q1, Q3) gestational age at delivery	39.4 weeks (38.1, 40.5)	38.5 weeks (36.6, 39.9)	38.6 weeks (36.1, 39.9)
% Delivery within 48 hours of the most severe HDP category	112/161 (69.6%)	84/118 (71.2%)	173/185 (93.5%)
% Induced labour‡	19/156 (12.2%)	14/106 (13.2%)	39/169 (23.1%)

* Pregnancies were classified by the most severe HDP category reached during pregnancy, and therefore each pregnancy only contributes to one category.

† For gestational hypertension, the timing of the gestational age at first and most severe HDP are equal.

‡ Excludes participants that had unknown labour induction status.

### Gestational hypertension

In the India cohort, gestational hypertension was associated with an increased risk of perinatal death (7.6% vs 2.1%; RR 3.53; 95% CI 1.48 to 8.43; p value 0.005) and stillbirth (6.1% vs 1.9%; RR 3.13; 95% CI 1.81 to 5.59; p value 0.02) as compared with no HDP, but was not associated with gestational age at delivery, preterm birth, SGA, birth weight or LBW (p value >0.05) (table 3). In the Tanzania cohort, gestational hypertension was also associated with an increased risk of perinatal death (9.1% vs 4.1%; RR 2.24; 95% CI 1.37 to 3.68; p value 0.001) and stillbirth (7.3% vs 2.7%; RR 2.67; 95% CI 1.52, 4.68; p value 0.001) as well as lower birth weight (mean difference −167 grams; 95% CI −259 to –76; p value <0.001) and increased risk of SGA (27.5% vs 21.7%; RR 1.34; 95% CI 1.02 to 1.75; p value 0.03), but was not associated with gestational age at delivery, preterm birth, or LBW (p value >0.05) (table 4).

**Table 3:** India cohort: association of gestational hypertension, pre-eclampsia without severe features and pre-eclampsia with severe features with perinatal outcomes (n=10 506 pregnancies with 10 570 foetuses)

	No hypertensive disorder of pregnancy(reference**)**	Gestational hypertension	Pre-eclampsia without severe features	Pre-eclampsia with severe features
Perinatal death				
n/N (%)	215/10180 (2.1%)	5/66 (7.6%)	13/183 (7.1%)	27/141 (19.1%)
Multivariable* relative risk (95% CI)	Ref.	3.53 (1.48, 8.43)	3.12 (1.82, 5.36)	8.60 (5.90, 12.53)
p-value		0.005	<0.001	<0.001
Stillbirth				
n/N (%)	189/10180 (1.9%)	4/66 (6.1%)	12/183 (6.6%)	23/141 (16.3%)
Multivariable* relative risk (95% CI)	Ref.	3.13 (1.81, 5.59)	3.18 (1.81, 5.59)	7.98 (5.28, 12.05)
p-value		0.02	<0.001	<0.001
Gestational age at delivery, weeks				
Mean±SD	38.9±1.9	38.7±1.7	38.2±2.2	37.3±2.7
Multivariable* mean difference (95% CI)	Ref.	−0.2 (–0.6, 0.2)	−0.7 (–1.0, –0.4)	−1.5 (–2.0, –1.0)
p-value		0.30	<0.001	<0.001
Preterm birth (<37 weeks gestation)				
n/N (%)	1143/9991 (11.4%)	10/62 (16.1%)	39/171 (22.8%)	52/118 (44.1%)
Multivariable* relative risk (95% CI)	Ref.	1.44 (0.82, 2.52)	1.89 (1.40, 2.55)	3.78 (3.04, 4.70)
p value		0.21	<0.001	<0.001
Small-for-gestational age birth (<10^th^ percentile)				
n/N (%)	3325/9991 (33.3%)	20/62 (32.2%)	61/171 (35.7%)	51/118 (43.2%)
Multivariable* relative risk (95% CI)	Ref.	1.06 (0.75, 1.51)	1.12 (0.91, 1.38)	1.44 (1.18, 1.74)
p value		0.74	0.29	<0.001
Birth weight, grams				
Mean±SD	2836±440	2839±528	2688±552	2447±718
Multivariable* mean difference (95% CI)	Ref.	−34 (−164, 95)	−169 (−253 to –85)	−413 (−542 to –284)
p value		0.60	<0.001	<0.001
Low birth weight (<2500 g)				
n/N (%)	1678/9991 (16.8%)	14/62 (22.6%)	47/171 (27.5%)	58/118 (49.2%)
Multivariable* relative risk (95% CI)	Ref.	1.51 (0.94, 2.39)	1.71 (1.32, 2.22)	3.21 (2.65, 3.90)
p value		0.08	<0.001	<0.001

* Multivariable model included covariates for maternal age (18–24, 24–29, ≥30 years), education (no formal education, primary/middle school, high school, pre-university college/diploma, or advanced), wealth quintile, height (<150, 150.0 to 154.9, ≥155 cm), body mass index (<18.5, 18.5–24.9, 25.0–29.9, ≥30 kg/m2) haemoglobin concentration (non-anaemia ≥11.0 g/dL, mild anaemia 10.0–10.9 g/dL, moderate or severe anaemia <10.0 g/dL) and randomised regimen (500 or 1500 mg calcium).

**Table 4:** Tanzania cohort: association of gestational hypertension, pre-eclampsia without severe features and pre-eclampsia with severe features with perinatal outcomes (n=10 299 pregnancies with 10 467 foetuses)

	No hypertensive disorder of pregnancy(reference)	Gestational hypertension	Pre-eclampsia without severe features	Pre-eclampsia with severe features
Perinatal death				
n/N (%)	408/9980 (4.1%)	15/165 (9.1%)	10/130 (7.7%)	32/192 (16.7%)
Multivariable* relative risk (95% CI)	Ref.	2.24 (1.37, 3.68)	1.98 (1.09, 3.59)	4.05 (2.91, 5.66)
p-value		0.001	0.01	<0.001
Stillbirth				
n/N (%)	272/9980 (2.7%)	12/165 (7.3%)	6/130 (4.6%)	28/192 (14.6%)
Multivariable* relative risk (95% CI)	Ref.	2.67 (1.52, 4.68)	1.74 (0.79, 3.81)	5.26 (3.64, 7.60)
p-value		0.001	0.17	<0.001
Gestational age at delivery, weeks				
Mean±SD	39.4±2.2	39.2±2.0	37.8±3.0	38.2±2.6
Multivariable* mean difference (95% CI)	Ref.	−0.2 (-0.5, 0.1)	−1.4 (-1.9, –0.9)	−1.2 (-1.6, –0.8)
p value		0.23	<0.001	<0.001
Preterm birth (<37 weeks gestation)				
n/N (%)	918/9708 (9.5%)	20/153 (13.1%)	43/124 (34.7%)	38/164 (23.2%)
Multivariable* relative risk (95% CI)	Ref.	1.25 (0.79, 1.97)	3.09 (2.29, 4.18)	2.44 (1.80, 3.29)
p value		0.34	<0.001	<0.001
Small-for-gestational age birth (<10^th^ percentile)				
n/N (%)	2100/9679 (21.7%)	42/153 (27.5%)	43/124 (34.7%)	46/164 (28.0%)
Multivariable* relative risk (95% CI)	Ref.	1.34 (1.02, 1.75)	1.53 (1.17, 2.01)	1.35 (1.05, 1.74)
p value		0.03	0.002	0.02
Birth weight, grams				
Mean±SD	3091±533	2953±577	2658±736	2759±685
Multivariable* mean difference (95% CI)	Ref.	−167 (−259 to –76)	−396 (−529 to –262)	−341 (−446 to –236)
p value		<0.001	<0.001	<0.001
Low birth weight (<2500 grams**)**				
n/N (%)	777/9679 (8.0%)	18/153 (11.8%)	45/124 (36.3%)	42/164 (25.6%)
Multivariable* relative risk (95% CI)	Ref.	1.52 (0.95, 2.42)	4.36 (3.27, 5.83)	3.32 (2.49, 4.44)
p value		0.08	<0.001	<0.001

* Multivariable model included covariates for maternal age (18–24, 24–29, ≥30 years), education (no formal education, primary, secondary or advanced), wealth quintile, height (<150 or ≥150 cm), body mass index (<18.5, 18.5–24.9, 25.0–29.9, ≥30 kg/m2) haemoglobin concentration (non-anaemia ≥11.0 g/dL, mild anaemia 10.0–10.9 g/dL, moderate or severe anaemia <10.0 g/dL), HIV status (positive or negative) and randomised regimen (500 or 1500 mg calcium).

### Pre-eclampsia without severe features

Pre-eclampsia without severe features was associated with an increased risk of perinatal mortality in India (7.1% vs 2.1%; RR 3.12; 95% CI 1.82 to 5.36; p value <0.001) and Tanzania (7.7% vs 4.1%; RR 1.98; 95% CI 1.09 to 3.59; p value 0.01). In both cohorts, pre-eclampsia without severe features was also associated with an increased risk of preterm birth and LBW as well as shorter duration of gestation and lower birthweights (p values <0.05). In Tanzania, there was also an association with an increased risk of SGA (34.7% vs 21.7%; RR 1.53; 95% CI 1.17 to 2.01; p value 0.002), but there was no association in India (35.7% vs 33.3%; RR 1.12; 95% CI 0.91 to 1.38; p value 0.29).

### Pre-eclampsia with severe features

The absolute risk of perinatal death for births to pregnant women with pre-eclampsia with severe features was quite high, 19.1% and 16.7% in the India and Tanzania cohorts, respectively. Pre-eclampsia with severe features was associated with a large magnitude of increased risk for perinatal death in India (RR 8.60; 95% CI 5.90 to 12.53; p value <0.001) and Tanzania (RR 4.05; 95% CI 2.91 to 5.66; p value <0.001) as compared with those without an HDP. Pre-eclampsia with severe features was also associated with increased risk of stillbirth, preterm birth, SGA and LBW as well as shorter gestation duration and lower birthweights in both settings (p value <0.05).

### Timing of hypertensive disorders of pregnancy onset

The association of early-onset (<34 weeks) as compared with late-onset HDP (≥34 weeks) with perinatal outcomes is presented in table 5 for both cohorts. Early-onset HDP was associated with an increased risk of perinatal death as compared with late-onset HDP in India (25.6% vs 5.2%; RR 4.79; 95% CI 2.68 to 8.54; p value <0.001) and Tanzania (36.9% vs 6.5%; RR 5.79; 95% CI 3.56 to 9.41; p value <0.001). Early-onset HDP was also associated with an increased risk of stillbirth and preterm birth as well as shorter gestation duration and lower birthweights in both cohorts (p value <0.05). In Tanzania, early-onset HDP was also associated with increased risk of SGA (38.6% vs 28.6%; RR 1.53; 95% CI 1.03 to 2.27; p value 0.04) and LBW (68.4% vs 17.2%; RR 5.14; 95% CI 3.72 to 7.09; p value <0.001), but there were no statistically significant associations found for these outcomes in India.

**Table 5:** Association of early-onset (<34 weeks gestation) as compared with late-onset (≥34 weeks gestation) hypertensive disorder of pregnancy with perinatal outcomes in India and Tanzania cohorts

	India		Tanzania	
	Early-onset hypertensive disorder of pregnancy(<34 weeks)*	Late-onset hypertensive disorder of pregnancy(≥34 weeks)*(reference)	Early-onset hypertensive disorder of pregnancy(<34 weeks)*	Late-onset hypertensive disorder of pregnancy(≥34 weeks)*(reference)
Perinatal death				
n/N (%)	31/121 (25.6%)	14/269 (5.2%)	31/84 (36.9%)	26/403 (6.5%)
Multivariable† relative risk (95% CI)	4.79 (2.68, 8.54)	Ref.	5.79 (3.56, 9.41)	Ref.
p-value	<0.001		<0.001	
Stillbirth				
n/N (%)	29/121 (24.0%)	10/269 (3.7%)	27/84 (32.1%)	19/403 (4.7%)
Multivariable† relative risk (95% CI)	6.20 (3.13, 12.27)	Ref.	7.06 (4.07, 12.26)	Ref.
p value	<0.001		<0.001	
Gestational age at delivery, weeks				
Mean±SD	37.0±3.2	38.3±1.8	34.7±3.7	39.0±1.9
Multivariable† mean difference (95% CI)	−1.3 (−2.0, −0.6)	Ref.	−4.4 (−5.3, −3.4)	Ref.
p value	<0.001		<0.001	
Preterm birth (<37 weeks gestation)				
n/N (%)	38/92 (41.3%)	63/259 (24.3%)	41/57 (71.9%)	60/384 (15.6%)
Multivariable† relative risk (95% CI)	1.64 (1.16, 2.32)	Ref.	5.58 (3.98, 7.81)	Ref.
p value	0.002		<0.001	
Small-for-gestational age (<10^th^ percentile)				
n/N (%)	36/92 (39.1%)	96/259 (37.1%)	22/57 (38.6%)	110/384 (28.6%)
Multivariable† relative risk (95% CI)	1.11 (0.81, 1.52)	Ref.	1.53 (1.03, 2.27)	Ref.
p value	0.52		0.04	
Birth weight, grams				
Mean±SD	2466±781	2693±547	2030±766	2912±581
Multivariable† mean difference (95% CI)	−239 (−410 to −67.7)	Ref.	−916 (−1116, −716)	Ref.
p value	0.006		<0.001	
Low birth weight (<2500 g)				
n/N (%)	38/92 (41.3%)	81/259 (31.3%)	39/57 (68.4%)	66/384 (17.2%)
Multivariable† relative risk (95% CI)	1.35 (0.99, 1.84)	Ref.	5.14 (3.72, 7.09)	Ref.
p value	0.06		<0.001	

* Includes gestational hypertension, pre-eclampsia without severe features and pre-eclampsia with severe features.

† Multivariable model included covariates for maternal age (18–24, 24–29, ≥30 years), education (India (no formal education, primary/middle school, high school, pre-university college/diploma or advanced); Tanzania (no formal education, primary, secondary or advanced)), wealth quintile, height (<150 or ≥150 cm), body mass index (<18.5, 18.5–24.9, 25.0–29.9, ≥30 kg/m2), haemoglobin concentration (non-anaemia ≥11.0 g/dL, mild anaemia 10.0–10.9 g/dL, moderate or severe anaemia <10.0 g/dL), HIV status (positive or negative) (Tanzania only) and randomised regimen (500 or 1500 mg calcium).

## Discussion

We used data from two large prospective studies with robust assessments of blood pressure and urine protein throughout pregnancy to examine the association of HDP with perinatal outcomes among nulliparous pregnancies without chronic hypertension in India and Tanzania. In both cohorts, we found that gestational hypertension, pre-eclampsia without severe features and pre-eclampsia with severe features were each associated with an increased risk of perinatal death as compared with pregnancies without an HDP. Pre-eclampsia with severe features, however, had the largest magnitude of risk for perinatal death in both settings. Pre-eclampsia with and without severe features was both independently associated with increased risk for preterm birth and LBW in both settings and was both also associated with greater risk for SGA births in the Tanzania cohort. In terms of timing of onset, early-onset HDP was associated with a strong magnitude of an increased risk for perinatal death, stillbirth, preterm birth and lower birthweights as compared with late-onset HDP in both cohorts.

Our findings confirm that pregnancies complicated by HDP are at greater risk for perinatal death, but we importantly specify that the magnitude of risk differs by HDP severity. While it was not surprising that HDP increased the risk of adverse perinatal outcomes, the magnitude of the risk in both relative and absolute terms was striking in our study cohorts, particularly for preeclampsia with severe features. Our findings also provide groups of HDP to focus prevention and treatment efforts to improve perinatal outcomes in the context of resource constraints. The most recent systematic review of pregnancy cohorts, which included limited data from Sub-Saharan Africa and South Asia, found that any HDP was associated with approximately three times the odds of perinatal death, and there was some indication that eclampsia, one of the clinical components of pre-eclampsia with severe features, may be associated with a greater magnitude of risk for perinatal death.^7^ However, this review did not consider HDP severity, which we found importantly modifies the magnitude of risk for perinatal death. In our cohorts, gestational hypertension and pre-eclampsia without severe features had similar magnitudes of risk for perinatal death within both India and Tanzania as compared with pregnancies without an HDP, respectively. Therefore, pregnancies with non-severe range hypertension (systolic blood pressure (BP) between 140 and 159 mm Hg or diastolic BP between 90 and 109 mm Hg), regardless of the presence of proteinuria, should both be considered at increased risk for perinatal death in resource-limited settings and require closer follow-up and management than pregnancies without hypertension. Pre-eclampsia with severe features was associated with a much larger magnitude of increased risk for perinatal death at ~8 times the risk of pregnancies without an HDP in India and ~4 times the risk in Tanzania. It is also important to note that the absolute risk of perinatal death was also quite high, with approximately one in five pregnancies complicated by pre-eclampsia with severe features experiencing a death in India and approximately one in six in Tanzania. Overall, our findings support that interventions that prevent or reduce the severity of HDP, including calcium supplementation^17^ and low-dose aspirin prophylaxis,^18^ may have important contributions to the attainment of global stillbirth and child mortality goals in resource-limited settings.

We also found that early-onset hypertensive disorders carried an increased risk of perinatal mortality and, not surprisingly, preterm birth, LBW, and most adverse perinatal outcomes as compared with late-onset HDP, since most pregnant women delivered within 48 hours of HDP diagnosis. Prior studies in high-income settings have also documented that early-onset HDP increases the risk of perinatal mortality.^19 20^ Early onset pre-eclampsia, particularly severe pre-eclampsia, poses management challenges since delivery may improve outcomes for the mother but may result in perinatal death in the absence of access to intensive care.^21^ However, clinical decisions that balance the risks of the mother and infant may be more complex in resource-limited settings, with limited resources for care for preterm or small newborns as well as mothers. Preterm birth is known to be a leading cause of stillbirth and neonatal death in resource-limited settings, and the relative magnitude of risk is larger as compared with high-income settings.^22^ Therefore, interventions, such as low-dose aspirin,^3 23^ that delay the onset of HDP may allow for greater time for foetal development and result in improved offspring survival, particularly in contexts with limited resources for intensive newborn care.

In addition, HDP categories were differentially associated with the risk of SGA and LBW in the study cohorts. Pre-eclampsia, particularly with early onset pre-eclampsia, is associated with suboptimal transformed spiral arteries in the first half of pregnancy that leads to reduced uteroplacental blood flow as the pregnancy advances, which can manifest as foetal growth restriction.^24 25^ Late-onset pre-eclampsia appears to have less of an association with placental dysfunction.^26^ In the Tanzania cohort, all three HDP categories and early-onset HDP were associated with an increased risk of SGA, an indicator of foetal growth restriction, while in India, only pre-eclampsia with severe features was associated with SGA, and there was no difference in risk of SGA by the timing of HDP onset. The proportion of SGA births was approximately twice as high in India as compared with Tanzania, which is consistent with global estimates.^22^ In this context, the relative contribution of HDP to SGA in India, where nutritional and other causes of foetal growth restriction may predominate, may be small. Additional research is needed on the epidemiology and mechanistic pathways through which HDP may affect foetal growth restriction in diverse resource-limited settings.

There are multiple mechanisms through which HDP severity and timing of onset may differentially increase the risk of perinatal mortality and other adverse birth outcomes. Placental insufficiency, when the placenta does not provide adequate oxygen and nutrients to the growing foetus, is associated with HDP and has been characterised as a contributor to suboptimal foetal growth as well as an increased risk of stillbirth.^27^,^29^ Hypertension in pregnancy is also a well-characterised risk factor for preterm labour due to multiple potential mechanisms including inflammation, oxidative stress and endocrine disruption, and severe range hypertension may exacerbate these processes.^30^,^32^ Further, pre-eclampsia with and without severe features, but not gestational hypertension, was associated with increased risks of preterm birth, a well-established risk factor for perinatal death.^11^ In both India and Tanzania cohorts, stillbirths accounted for the majority of perinatal deaths. This finding further supports the urgent need for strategies to reduce stillbirth globally and indicates that interventions for the prevention and treatment of HDP may play an important role in reaching global stillbirth goals in LMICs.^9^

Our study had several strengths including the large size of the cohorts, rigorous measurement of blood pressure and proteinuria, and the use of ultrasound for gestational age dating. Nevertheless, there were several limitations to our study. The study cohorts included only nulliparous women as this was an inclusion criterion for the parent calcium supplementation trials. We excluded pregnant women with chronic hypertension since the prevalence was low (<1% in both cohorts) and therefore did not afford statistical power to examine perinatal outcomes. The parent trials in India and Tanzania enrolled nulliparous pregnant women, and therefore most participants were young, which contributed to the low risk of chronic hypertension. We also did not evaluate the role of HDP treatment in perinatal outcomes. In our study, pregnant women received standard-of-care treatment within the healthcare system, and we plan to evaluate the association of timing and type of HDP treatment with perinatal outcomes in a future study. In addition, our analyses focused on the role of HDP in offspring outcomes, further studies are needed to characterise maternal outcomes in HDP in diverse settings. Finally, our study cohorts enrolled nulliparous pregnant women from urban settings in Tanzania and India, and therefore care should be taken when generalising our study findings to other pregnant populations and settings. As seen in our study, there were some differences in the magnitude of risks for HDP between the two cohorts, which supports that there may be important underlying differences between populations.

## Conclusion

Overall, we found that all three HDP categories were associated with an increased risk for perinatal death, but the magnitude of the association differed by the severity and timing of HDP diagnosis. Notably, pre-eclampsia with severe features had increased risk for nearly all perinatal outcomes examined, including a particularly high magnitude of risk for perinatal death. These findings suggest that interventions that prevent, reduce the severity or delay the onset of HDP may significantly improve perinatal outcomes in LMICs. There are a limited number of interventions known to reduce the risk or delay HDP, including calcium supplementation and low-dose aspirin prophylaxis, and implementation research is needed to understand how to deliver these interventions effectively at scale in LMICs. Research is also necessary to develop new interventions to reduce the burden of HDP as well as identify biomarker predictors of early-onset HDP to inform clinical care.

## Supplementary material

10.1136/bmjgh-2024-016339 online supplemental file 1

10.1136/bmjgh-2024-016339 online supplemental file 2

## Data Availability

Data are available upon reasonable request.
