# Supplementary material for: Hypertensive disorders of pregnancy and perinatal outcomes: two prospective cohort studies of nulliparous women in India and Tanzania
Source: BMJ Glob Health. 2025 Jul 10;10(7):e016339. doi: 10.1136/bmjgh-2024-016339 (PMC12258372; doi:10.1136/bmjgh-2024-016339)
Supplement: online supplemental file 1 [file bmjgh-10-7-s001.pdf]

Supplemental File 1 – Reflexivity Statement

1. How does this study address local research and policy priorities?

This study addresses the burden and consequences of hypertensive disorders of pregnancy, which are among the leading causes of maternal and child morbidity and mortality in South Asia and sub-Saharan Africa. As a result, understanding differences in the etiology of hypertensive disorder phenotypes and their differences in the magnitude of association with fetal and infant outcomes is important for program planning and intervention design in India, Tanzania, and other resource-limited settings.

2. How were local researchers involved in study design?

The multi-disciplinary team in India, Tanzania, and the US partners developed the design of the parent trial protocol together in partnership. The team worked together to design the protocols for assessing hypertension and proteinuria, as well as for fetal and infant assessment. These protocols took into local standard of care and the need for enhanced measurement in a research study. For this, the hypertensive disorders phenotype paper, the study team developed the research question and the exposure and outcomes of interest on weekly study calls between the India, Tanzania, and US study teams. The statistical analysis plan was finalized at a meeting in Boston in 2023 that was attended by members from each study team. Researchers from India and Tanzania contributed actively to all steps of the study design, analyses, and writing.

3. How are research staff who conducted data collection acknowledged?

We have included the study staff who collected the data in the acknowledgements section of the manuscript.

4. Do all members of the research partnership have access to study data?

All members of the partnership have access to data and statistical analysis code.

5. How was data used to develop analytical skills within the partnership?

The analysis plan was developed by all members, and this manuscript was developed at a workshop conducted in Boston in June 2023 that was attended by the India, Tanzania, and US team members. At this meeting, the statistical code was refined, and the results were interpreted by all members.

6. How have research partners collaborated in interpreting study data?

There was an inclusive process during the development of the statistical analysis plan, implementation of the data analysis, and interpretation of the results. Primarily, this was done at the June 2023 analysis meeting that was attended by team members from India and Tanzania in addition to US counterparts. After the meeting, the interpretations were strengthened by all team members through the finalization of the manuscript draft.

7. How will research products be shared to address local needs?

The study results will be shared through dissemination activities with the local, regional, and national authorities in India and Tanzania.

8. **How is the leadership, contribution and ownership of this work by LMIC researchers recognised within the authorship?**

The co-first authors are from Tanzania and India, respectively.

9. **How have early career researchers across the partnership been included within the authorship team?**

Of the authors, 50% of the authors (9 individuals) are junior faculty and research staff.

10. **How has gender balance been addressed within the authorship?**

There are 18 authors on the manuscript – 8 are female and 10 are male.

11. **How has the project contributed to training of LMIC researchers?**

The authorship team is composed of senior and junior faculty as well as data team members from India, Tanzania, and the US. The process of developing the statistical analysis, implementing the data analysis, and interpreting the data included all individuals. LMIC researchers enhanced their skills in design, analysis, and writing as part of their participation.
