# Supplementary material for: Hypertensive disorders of pregnancy and perinatal outcomes: two prospective cohort studies of nulliparous women in India and Tanzania
Source: BMJ Glob Health. 2025 Jul 10;10(7):e016339. doi: 10.1136/bmjgh-2024-016339 (PMC12258372; doi:10.1136/bmjgh-2024-016339)

Supplemental Figure 1. India study participant flow chart

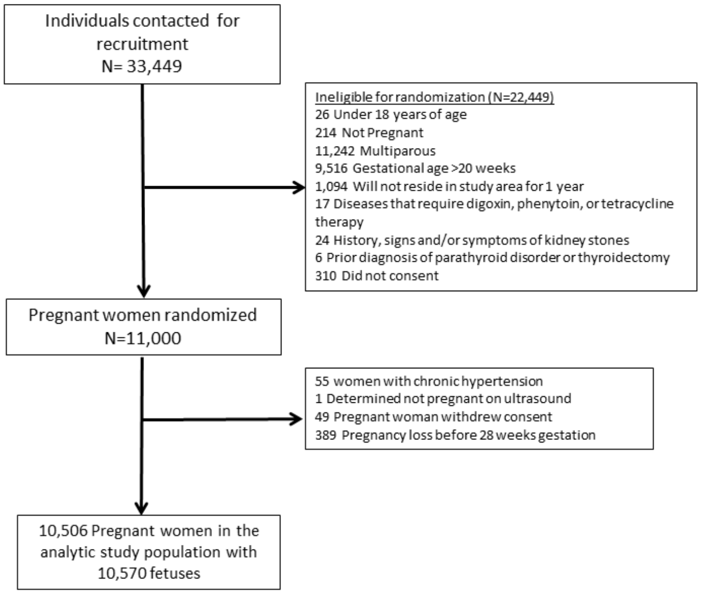

Supplemental Figure 2. Tanzania study participant flow chart

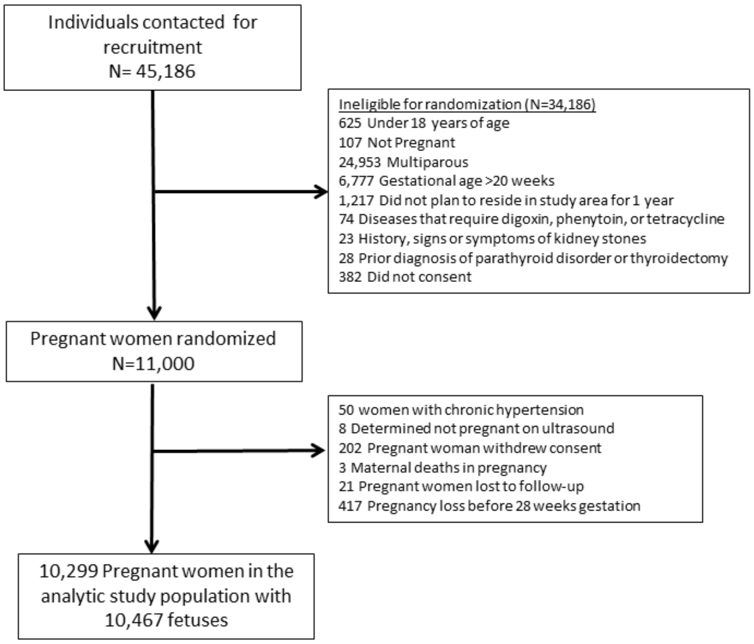

Supplement: online supplemental file 2 [file bmjgh-10-7-s002.pdf]
